# Pharmacokinetic Study of Four Major Bioactive Components of Liandan Xiaoyan Formula in Ulcerative Colitis and Control Rats Using UPLC-MS/MS

**DOI:** 10.3389/fphar.2022.936846

**Published:** 2022-07-04

**Authors:** Kaihui Zhang, Zenghui Lu, Qian Wang, Fangle Liu, Meiqi Wang, Chaozhan Lin, Chenchen Zhu

**Affiliations:** ^1^ School of Pharmaceutical Sciences, Guangzhou University of Chinese Medicine, Guangzhou, China; ^2^ School of Basic Medical Sciences, Guangzhou University of Chinese Medicine, Guangzhou, China

**Keywords:** Liandan Xiaoyan Formula, pharmacokinetic, ulcerative colitis, bioactive component, UPLC-MS/MS

## Abstract

Liandan Xiaoyan Formula (LXF), a classic Traditional Chinese medicine (TCM) formula, is composed of two Chinese herbal medicines for treating bowel disease under the TCM theory. This study aimed to develop a rapid, stable, sensitive, and reliable method based on ultra-high performance liquid chromatography-tandem mass spectrometry (UPLC-MS/MS) to simultaneously determine four major bioactive components of LXF (andrographolide, dehydroandrographolide, 1-methoxicabony-β-carboline, 4-methoxy-5-hydroxy-canthin-6-one) in rat serum and evaluate the pharmacokinetic characteristics of LXF in ulcerative colitis (UC) and control rats. After pretreating by protein precipitation with methanol, separation was performed on a UPLC C18 column using gradient elution with a mobile phase consisting of acetonitrile and 0.1% formic acid at a flowing rate of 0.4 ml/min. Detection was performed on Triple-TOF™ 5600 mass spectrometry set at the positive ionization and multiple reaction monitoring (MRM) mode. The validated method showed good linearity (*R* ^2^ ≥ 0.9970), the intra- and inter-day accuracy were within ±11.58%, whereas the intra- and inter-day precision were less than 13.79%. This method was validated and applied to compare the pharmacokinetic profiles of the analytes in serum of UC induced by dextran sulphate sodium (DSS) and control rats after oral administration of LXF. The results showed that four major bioactive components of LXF were quickly absorbed after oral administration in both groups, with higher exposure levels in the UC group. This relationship between the active ingredients’ pharmacokinetic properties provided essential scientific information for applying LXF in clinical.

## 1 Introduction

Ulcerative colitis (UC) is an idiopathic, relapsing, chronic inflammatory bowel disease (IBD) that occurs in the colon and rectum (Feuerstein et al., 2019; Sun et al., 2021). Some studies suggested that it was related to inappropriate immune responses to enteric commensal microbes in genetically susceptible individuals exposed to environmental risk factors (Ng et al., 2013; Du and Ha, 2020). Inadequate treatment of UC might result in continuous bowel damage and subsequently increased risks of hospitalizations and colorectal cancer (Ungaro et al., 2017). Since the mid-20th century, the incidence and prevalence of UC have been rising (Molodecky et al., 2012; Mak et al., 2020). Millions of people worldwide are affected by UC and considering morbidity and mortality related to UC, societal costs are substantial (Cohen et al., 2010). Until today a specific cure for UC has not been found, and the treatments available are limited to alleviation. Conventional treatment for UC is combinations of pharmacologic agents, such as azathioprine, aminosalicylates, and corticosteroids to promote alleviation, prevention of relapses, and mucosal healing. However, using these drugs in UC is associated with numerous systemic and local individual adverse effects, such as headache, nausea, loss of appetite, vomiting, and rash (Roselli and Finamore, 2020). Therefore, developing novel and effective therapeutic agents for UC is urgently needed.

**FIGURE 1:**
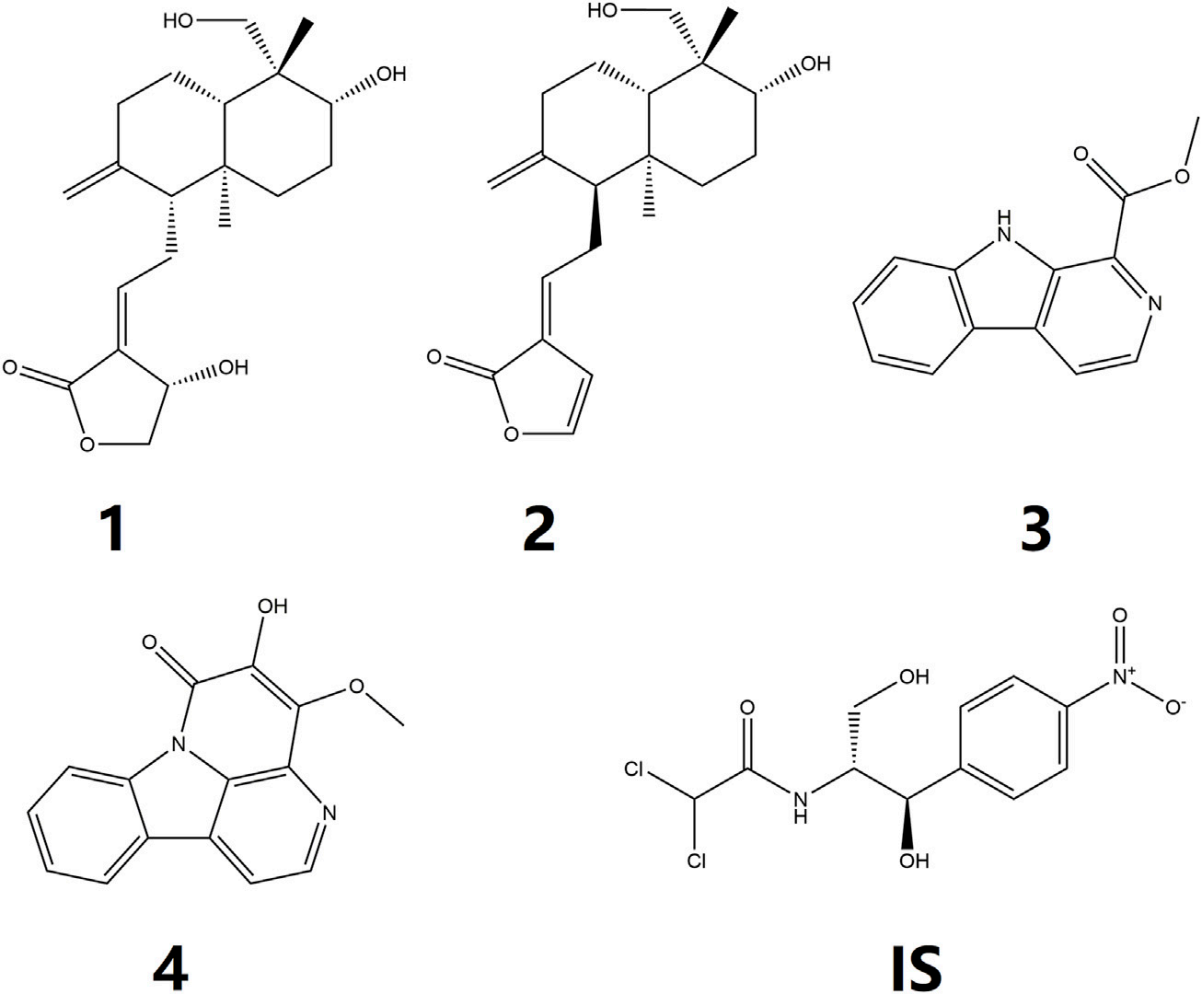
Chemical structures of **1** andrographolide, **2** dehydroandrographolide, **3** 1-methoxicabony-β-carboline, **4** 4-methoxy-5-hydroxy-canthin-6-one, and chloramphenicol (**IS**).

**FIGURE 2:**
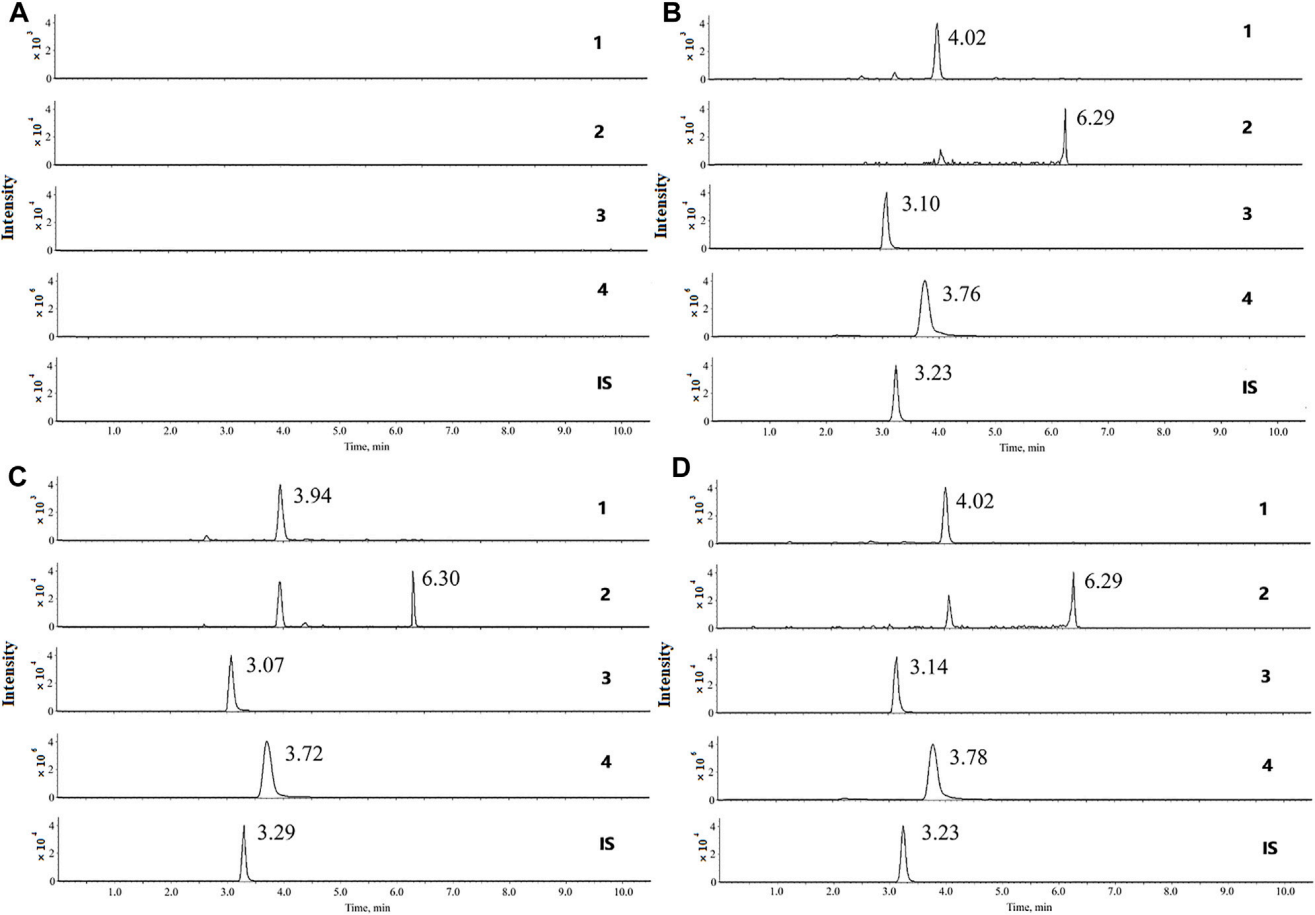
Multiple reaction monitoring (MRM) chromatograms of the four compounds and chloramphenicol (IS) in rats. **(A)** blank serum; **(B)** blank serum with standard substance and IS; **(C)** serum of control group; **(D)** serum of UC group. **1** andrographolide, **2** dehydroandrographolide, **3** 1-methoxicabony-β-carboline, **4** 4-methoxy-5-hydroxy-canthin-6-one, and chloramphenicol (**IS**).

Traditional Chinese medicine (TCM) has been refined for thousands of years *via* its continued clinical application (Ma et al., 2016; Zhang et al., 2021). TCM stresses mixtures with more than one herb extract, and the synergistic effect of various components in these herbs produces high efficacies (Yang et al., 2016). Liandan Xiaoyan Formula (LXF), a classic TCM formula, is composed of two Chinese herbal medicines that are Herba andrographis [dried herb of *Andrographis paniculata* (Burm. f.) Nees, also called *Chuanxinlian*], and Ramulus et folium picrasmae [dried twigs and leaves of *Picrasma quassioides* (D. Don) Benn, also called *Kumu*], and this classic formula has been processed into many kinds of patent medicine with the efficiency of damp-heat dysentery, anti-bacterial, anti-inflammatory, and clear away heat and toxic material, and have been widely used for the treatment of various intestinal inflammation in clinic. Chemical components of the two herbal medicines in LXF have been reported to have several biological activities associated with their beneficial efficacy. Phytochemical research has exposed that diterpene lactones, such as andrographolide (Figure 1) and dehydroandrographolide (Figures 1, 2), were the main bioactive components of *Chuanxinlian* (Sareer et al., 2014). The diterpene lactones possess anti-inflammatory (Burgos et al., 2020), hepatoprotective (Patil and Jain, 2021), cardioprotective (Mehta et al., 2022), and immunostimulant, anti-oxidant, anti-viral, anti-bacterial, gastroprotective activities (Wasman et al., 2011; Arifullah et al., 2013). *Kumu* has numerous alkaloids as the main active components, which were reported to have effects on various inflammatory and infectious diseases, such as acute tonsillitis, diarrhea.

**FIGURE 3:**
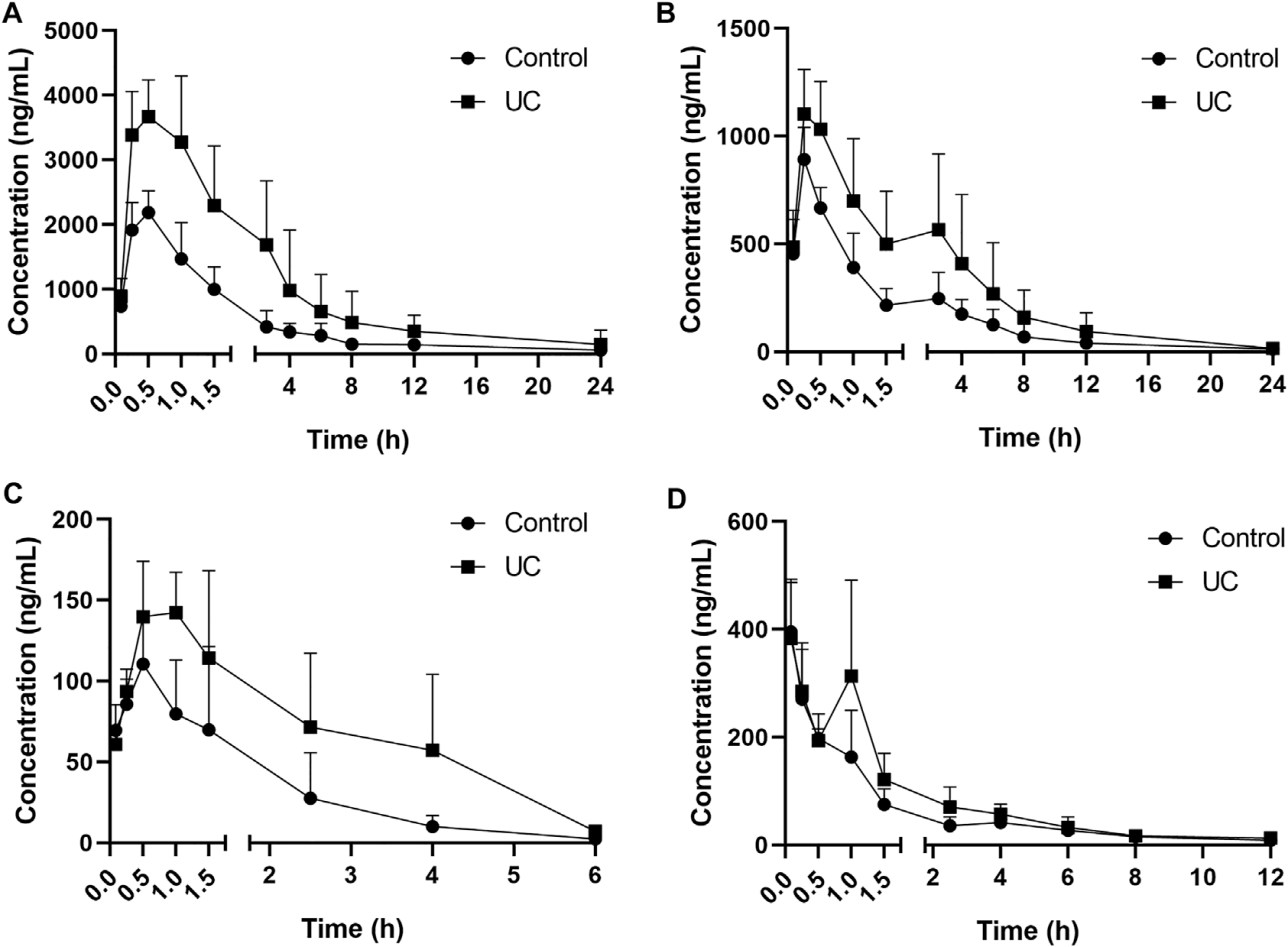
Mean serum concentration–time curves for **(A)** andrographolide, **(B)** dehydroandrographolide, **(C)**, 1-methoxicabony-β-carboline, **(D)** 4-methoxy-5-hydroxy-canthin-6-one (*n* = 8).

Pneumonia, and acute upper respiratory tract infections (Wang et al., 2018; Hu et al., 2021). Our previous study indicated that 1-methoxicabony-β-carboline (Figures 1–3), 4-methoxy-5-hydroxy-canthin-6-one (Figures 1–4) were the two major bioactive alkaloids in Kumu (Zhang et al., 2020). Therefore, this study focuses on these four compounds.

The pharmacokinetic characteristics of the active compounds in TCM have been used to evaluate clinical efficacy, guide the rational use of herbal medicines, and promote their development (Zhang et al., 2018; Zhu et al., 2020). These days, ultra-high performance liquid chromatography coupled with electrospray ionization tandem quadrupole/time of flight mass spectrometry (UPLC-MS/MS) has been used as a general approach for metabolic and pharmacokinetic profiling study of natural products (Yao et al., 2018; Li et al., 2019; Wu et al., 2020). Recent research has also shown that the internal environment could influence the pharmacokinetics of drugs in a pathological state, such as colitis (Liu et al., 2020). It is indicated that pharmacokinetic study of XLF in UC state is necessary to provide a basis for clinical use. Therefore, we decided to investigate the pharmacokinetic profiles of LXF *in vivo* between ulcerative colitis and normal physiological condition in this research.

In this study, we established a stable, sensitive, and reliable UPLC-MS/MS method for quantitative analysis of the concentrations of four major active compounds of LXF in rat serum. This method has been applied and validated in a comparative pharmacokinetic study after oral administration of XLF to UC and control rats.

## 2 Materials and Methods

### 2.1 Chemicals and Reagents

Dextran sulphate sodium (DSS) (Lot number D7002900) was obtained from Yeasen Biotechnology (shanghai) Co., Ltd (Shanghai, China). Andrographolide (Lot number 110797-201609) and dehydroandrographolide (Lot number 110854-201710) were purchased from National Institutes for Food and Drug Control (Beijing, China). 1-methoxicabony-β-carboline and 4-methoxy-5-hydroxy-canthin-6-one were purified and identified using Infrared Spectroscopy (IR), UV–visible spectroscopy (UV), mass spectrometry (MS), and Nuclear Magnetic Resonance Spectroscopy (NMR) as described in our previous studies, and the purity of them was more than 98% (Yang et al., 2016; Liu et al., 2020; Zhang et al., 2021). Chloramphenicol (Lot number Y27M6C2), internal standard (IS), was purchased from Shanghai Yuanye Bio-Technology Co., Ltd (Shanghai, China). MS-grade methanol, acetonitrile, and formic acid were obtained from Merck Co., Ltd (Darmstadt, Germany). Deionized water was purified using the Milli-Q system (Millipore, Milford, MA, United States) and used for the entire study. Furthermore, all other reagents were analytical grade from Guangzhou Chemical Reagent Co., Ltd (Guangzhou, China).

### 2.2 Liandan Xiaoyan Formula Extract Preparation

The preparation and intragastric dose of the LXF sample followed our previous research (Lu et al., 2021). Meanwhile, the quality of LXF was controlled according to the contents of four main components 69.78 mg/g of andrographolide, 38.17 mg/g of dehydroandrographolide, 0.79 mg/g of 1-methoxicabony-β-carboline, and 5.68 mg/g of 4-methoxy-5-hydroxy-canthin-6-one (Supplementary Material Part A).

### 2.3 Animals, Dosing, and Sampling

Sixteen male Sprague-Dawley (SD) rats (220 ± 10 g) were provided from the Liaoning Changsheng Biotechnology Co., Ltd (Benxi, China; with license No. of SCXK, 2020-0001). The rats were kept in an environment (22 ± 2°C, relative humidity 50 ± 5%) under a 12-h light-dark cycle with a free diet and water for 3 days. All experiments on animals were conducted strictly under the related ethics regulations of the Committee on Use and Care of Animals of Guangzhou University of Chinese Medicine (Guangzhou, China) (No. 712052).

All rats were assigned equally and randomly to the control and model groups. The rats of the model group were free to access 4% DSS for 7 days to induce UC, and rats of the control group were given pure water (Chen et al., 2020). Before the pharmacokinetics study, the DSS-induced UC rats (model) were established successfully (Supplementary Material Part B).

After fasting for 12 h, the rats were orally administered LXF at a 3.30 g/kg dose according to Prescription preparation of Chinese medicine, volume 10 (Lu et al., 2021). Serum samples (approximately 0.5 ml) were collected from the fosse orbital vein using 1.5 ml polythene tubes before the administration and at 0.083, 0.25, 0.5, 1, 1.5, 2.5, 4, 6, 8, 12, 24, 36, and 48 h after dosing and centrifuged immediately at 3,500 rpm for 15 min. Serum was transferred into clean tubes and stored at −80°C.

### 2.4 Sample Pretreatment

100 μl of IS solution (960 ng/ml) was added to a 100 μl serum sample and vortex-mixed for 0.5 min, and then 800 μl of methanol was added and vortex-mixed for 3 min. After centrifugation at 12,000 rpm for 15 min (4°C), the supernatant of the mixture was transferred to another vial and evaporated to dryness with nitrogen purging. The residue was resolved with 100 μ of methanol, vortex-mixed for 1 min, and centrifuged at 12,000 rpm for 10 min (4°C). A 2 μl aliquot of the supernatant was injected into the UPLC-MS/MS system for analysis.

### 2.5 Preparation of Standard and Quality Control Samples

Standard stock solutions of andrographolide (5,125 ng/ml), dehydroandrographolide (2000 ng/ml), 1-methoxicabony-β-carboline (576 ng/ml), 4-methoxy-5-hydroxy-canthin-6-one (525 ng/ml), and IS (1,000 ng/ml) were prepared in methanol. Then, the calibration standard stock solutions of gradient concentrations were gained by serial dilution. The calibration standards were prepared by spiking 100 µl of appropriate standard working solution into 100 µl of blank serum to obtain the concentrations ranging from 5,125.00 to 51.25 ng/ml for andrographolide, 2000.00–20.00 ng/ml for dehydroandrographolide, 576.00–2.30 ng/ml for 1-methoxicabony-β-carboline, 525.00–2.10 ng/ml for 4-methoxy-5-hydroxy-canthin-6-one.

Quality control (QC) samples at low, middle and high concentrations (61.50, 384.38, and 4,000.00 ng/ml for andrographolide; 24.00, 150.00, and 1,500.00 ng/ml for dehydroandrographolide; 6.91, 43.20, and 450.00 ng/ml for 1-methoxicabony-β-carboline; 6.30, 39.38, and 400.00 ng/ml for 4-methoxy-5-hydroxy-canthin-6-one) were also prepared by the same procedures.

### 2.6 Instrumentation and Chromatographic Conditions

Chromatographic analysis was performed using UPLC-MS/MS, utilizing a Finnigan Surveyor auto-sampler and a Finnigan Surveyor LC pump combined with a triple quadrupole TSQ Quantum mass spectrometer electrospray ionization (ESI) interface (Thermo Fisher, Palo Alto, CA). The chromatographic separation was performed on the Waters Acquity UPLC BEH C18 column (2.1 mm × 100 mm; 1.7 µm). The mobile phase consisted of acetonitrile (A) and 0.1% formic acid (B) with a gradient elution program (0-5 min, 20% A-35% A; 5-6 min, 35% A-100% A; 6–8min, 100% A; 8–8.5 min, 100% A-20% A; 8.5–10.5 min, 20% A) at a flow rate of 0.4 ml/min. The column temperature was maintained at 40°C throughout the analysis. The injection volume was 2 μl.

Quantification was performed using multiple reaction monitoring (MRM) in the positive ionization mode, and the optimized MRM parameters of the four analytes and IS are listed in Table 1. The MRM transition, retention time (RT), and collision energy (CE) values were based on Thermo Foundation 2.0. Curtain gas pressure at 35 psi, ion source gas 1 and gas 2 at 55 psi, ion spray voltage at 5500 V, and temperature at 500°C.

**TABLE 1:** The mass spectrometry parameters of tested compounds.

No.	Compounds	RT (min)	(MRM)*m/z*	CE (eV)
1	Andrographolide	3.94	351.22→257.15	15
2	Dehydroandrographolide	6.30	333.21→257.15	15
3	1-methoxicabony-β-carboline	3.07	227.08→195.05	25
4	4-methoxy-5-hydroxy-canthin-6-one	3.72	267.08→252.05	35
IS	Chloramphenicol	3.29	323.02→275.00	20

### 2.7 Method Validation

Specificity was assessed by analyzing blank serum samples to investigate the potential interferences of compounds and IS. The calibration curves for quantitative analysis were drawn by plotting the peak area ratio (y) of each compound to IS against the corresponding nominal concentration (x) using weighted (1/x^2^) least-squares linear regression. Simultaneously, the lowest concentration in the calibration curve was defined as the lower limit of quantification (LLOQ) (S/N ≥ 10). The precision and accuracy were evaluated using six replicates of QC samples on three consecutive days. The intra- and inter-day accuracy and precision variations were expressed as the relative error (RE) and relative standard deviation (RSD).

The stability test of QC samples in rat serum was assessed in a different store environment, including at room temperature (25°C) for 24 h, at −80 °C for 1 month, and three freeze-thaw cycles. Recovery was determined at three QC levels and calculated by comparing the compound standard peak areas obtained from extracted samples with post-extracted samples spiked with the compound. Matrix effects were calculated by matching spiking post-extracted blank serum samples with corresponding standard clean solutions at three concentrations of QC samples.

### 2.8 Statistical Analyses

Non-compartment model pharmacokinetic parameters, including the time to reach the maximum serum concentration (T_max_), the maximum serum concentration (C_max_), the elimination half-time (T_1/2_), the area under the serum concentration-time curve (AUC), and mean residence time (MRT), were calculated by DAS (version 3.2.8, Chinese Pharmacological Association, Anhui, China). The difference between the control and model groups was statistically analyzed using SPSS 26.0 (IBM Company, Chicago). All data in this study were subjected to a one-way analysis of variance (ANOVA) and then expressed by mean ± standard deviation (SD). LSD *t*-test was applied when the homogeneity of variance assumptions was satisfied; otherwise, Dunnett’s *t*-test was used. A *p* < 0.05 was considered statistically significant.

## 3 Results and Discussion

### 3.1 Method Validation

#### 3.1.1 Specificity

As shown in Figure 2, no endogenous interference was observed in blank serum, which proves the assay specificity.

#### 3.1.2 Calibration Curves and Lower Limit of Quantification

All calibration curves showed well linearity, and the coefficient of determination (*R* ^2^) was greater than 0.9970. Table 2 lists regression equations, correlation coefficients, and linear ranges of the four analytes.

**TABLE 2:** The details of the regression analysis of four compounds in rat serum.

Compounds	Calibration curves	*R* ^2^	Liner range (ng/ml)	LLOQ (ng/ml)
Andrographolide	Y = 0.0001X + 0.0056	0.9998	51.25–5,125.00	51.25
Dehydroandrographolide	Y = 0.0001X + 0.0068	0.9970	20.00–2000.00	20.00
1-methoxicabony-β-carboline	Y = 0.0445X + 0.1443	0.9981	2.30–576.00	2.30
4-methoxy-5-hydroxy-canthin-6-one	Y = 0.0682X + 0.1070	0.9989	2.10–525.00	2.10

#### 3.1.3 Accuracy and Precision

As shown in Table 3, intra- and inter-day accuracy were within ± 11.58%, whereas intra- and inter-day precision were less than 13.79%, indicating that these two parameters were all within the acceptable range for research in biological media.

**TABLE 3:** |Precision and accuracy of four compounds in rat serum (*n* = 6).

Compounds	Concentration (ng/ml)	Accuracy (RE, %)		Precision (RSD, %)	
		Intra-day	Inter-day	Intra-day	Inter-day
Andrographolide	61.50	−1.46	−0.75	9.31	12.74
	384.38	11.58	3.52	6.77	9.59
	4,000.00	5.81	6.70	9.76	8.03
Dehydroandrographolide	24.00	4.76	−11.05	12.12	11.62
	150.00	7.25	4.30	4.30	9.57
	1,500.00	3.62	−1.05	3.91	8.62
1-methoxicabony-β-carboline	6.91	4.94	3.11	4.05	3.84
	43.20	3.24	1.52	4.23	4.49
	450	4.13	3.46	5.82	7.96
4-methoxy-5-hydroxy-canthin-6-one	6.30	−4.76	1.74	13.79	9.51
	39.38	2.62	7.96	2.84	4.71
	400.00	4.72	5.01	6.59	6.63

#### 3.1.4 Recovery and Matrix Effect

As shown in Table 4, the RSD values of extraction recovery and matrix effect were not greater than 10.15%, which indicated that recoveries were consistent and reproducible at different concentrations, and no significant matrix effects were observed for the four analytes.

**TABLE 4:** Recovery and matrix effects of the four compounds in rat serum (*n* = 6).

Compounds	Concentration (ng/ml)	Recovery		Matrix effect	
		Mean ± SD (%)	RSD (%)	Mean ± SD (%)	RSD (%)
Andrographolide	61.50	91.98 ± 5.07	5.51	69.98 ± 7.99	11.42
	384.38	82.03 ± 3.83	4.67	69.77 ± 2.24	3.21
	4,000.00	86.51 ± 2.09	2.42	72.36 ± 2.83	3.91
Dehydroandrographolide	24.00	65.29 ± 2.86	4.37	73.18 ± 7.43	10.15
	150.00	66.86 ± 4.03	6.03	75.50 ± 4.29	5.68
	1,500.00	66.41 ± 3.03	4.56	75.63 ± 3.55	4.69
1-methoxicabony-β-carboline	6.91	91.51 ± 0.74	0.81	109.73 ± 1.20	1.09
	43.20	91.84 ± 0.88	0.96	112.67 ± 2.41	2.14
	450.00	92.18 ± 1.21	1.31	108.91 ± 3.06	2.81
4-methoxy-5-hydroxy-canthin-6-one	6.30	80.78 ± 2.55	3.15	112.25 ± 2.10	1.87
	39.38	78.69 ± 1.48	1.88	112.36 ± 2.19	1.95
	400.00	82.74 ± 2.11	2.56	106.75 ± 2.25	2.11

#### 3.1.5 Stability

As shown in Table 5, the four analytes in rat serum under different conditions were stable, and their RSD was less than 10.85%.

**TABLE 5:** The stability test of four compounds in rat serum (*n* = 6).

Compounds	Concentration (ng/ml)	25°C for 12 h		4°C for 24 h		−80°C for 30 days		Three freeze-thaw	
		Mean (ng/ml)	RSD (%)	Mean (ng/mL)	RSD (%)	Mean (ng/ml)	RSD (%)	Mean (ng/mL)	RSD (%)
Andrographolide	61.50	56.68 ± 4.20	7.41	58.54 ± 4.47	7.64	58.97 ± 2.93	4.97	60.13 ± 3.34	5.55
	384.38	399.59 ± 9.79	2.45	383.22 ± 9.64	2.51	370.23 ± 17.30	4.67	396.68 ± 13.05	3.29
	4,000.00	3,896.61 ± 129.89	3.33	3,915.74 ± 98.45	2.51	3,904.68 ± 126.18	3.23	3,923.07 ± 110.39	2.81
Dehydroandrographolide	24.00	23.51 ± 0.69	2.92	25.71 ± 1.69	6.59	22.46 ± 2.57	11.45	21.20 ± 2.30	10.85
	150.00	153.93 ± 10.00	6.50	156.59 ± 14.58	9.31	151.06 ± 3.31	2.19	143.60 ± 9.61	6.69
	1,500.00	1,439.13 ± 52.46	3.64	1,421.52 ± 61.75	4.34	1,419.63 ± 51.60	3.63	1,428.09 ± 67.73	4.74
1-methoxicabony-β-carboline	6.91	7.09 ± 0.16	2.19	7.71 ± 0.12	1.49	7.43 ± 0.66	8.83	6.63 ± 0.51	7.63
	43.20	42.21 ± 3.37	7.98	44.64 ± 3.27	7.32	42.66 ± 1.92	4.49	42.04 ± 2.18	5.18
	450.00	439.66 ± 16.78	3.82	442.09 ± 15.98	3.61	436.91 ± 13.57	3.11	440.84 ± 17.31	3.93
4-methoxy-5-hydroxy-canthin-6-one	6.30	5.50 ± 0.37	6.82	6.38 ± 0.41	6.38	6.39 ± 0.23	3.64	5.65 ± 0.12	2.11
	39.38	40.41 ± 3.15	7.78	39.35 ± 4.12	10.47	42.93 ± 1.44	3.35	41.43 ± 0.76	1.83
	400.00	387.02 ± 9.84	2.54	391.53 ± 10.08	2.57	381.78 ± 17.23	4.51	386.59 ± 20.07	5.19

### 3.2 Pharmacokinetics

The UPLC method was applied to a comparative pharmacokinetic study of four compounds in control and UC rat serum after oral administration of LXF. The concentration-time curves of the compounds in the control and model groups are presented in Figure 3, and the corresponding pharmacokinetic parameters are shown in Table 6.

**TABLE 6:** The main pharmacokinetic parameters of the four compounds in rat serum after oral administration of XLF (*n* = 8).

Compounds	Group	C_max_ (ng/L)	T_max_ (h)	T_1/2_ (h)	MRT_0→∞_ (h)	AUC_0→t_ (ng/mL*h)	AUC_0→∞_ (ng/mL*h)
andrographolide	Control	2,309.75 ± 372.63	0.53 ± 0.21	8.77 ± 2.96	8.42 ± 2.75	6,430.54 ± 1,111.78	7,120.49 ± 1,388.81
	UC	4,093.93 ± 493.79^b^	0.69 ± 0.26	7.40 ± 5.02	7.40 ± 5.02	15,854.73 ± 8,467.51^b^	18,431.87 ± 12,812.25^a^
dehydroandrographolide	Control	892.35 ± 147.60	0.28 ± 0.09	2.28 ± 1.69	5.04 ± 1.96	2,327.38 ± 512.51	2,355.73 ± 560.39
	UC	1,152.99 ± 200.01^a^	0.38 ± 0.13	2.86 ± 2.01	5.08 ± 1.53	4,694.04 ± 2,258.15^a^	4,794.15 ± 2,389.47^a^
1-methoxicabony-β-carboline	Control	128.95 ± 33.92	0.69 ± 0.37	1.02 ± 0.43	1.52 ± 0.33	214.95 ± 78.40	218.79 ± 79.21
	UC	159.58 ± 18.88^a^	1.13 ± 0.23^a^	1.20 ± 0.41	2.23 ± 0.63^a^	424.43 ± 143.29^b^	444.08 ± 153.98^b^
4-methoxy-5-hydroxy-canthin-6-one	Control	395.26 ± 97.67	0.08 ± 0.00	3.15 ± 0.96	3.59 ± 1.43	553.61 ± 74.16	594.78 ± 117.49
	UC	419.23 ± 103.53	0.43 ± 0.47	3.30 ± 1.52	3.66 ± 1.55	758.79 ± 142.16^b^	817.12 ± 177.20^a^

a *p* < 0.05 and.

b *p* < 0.01 compared with the control group.

Compared with the control group, the AUC_0–t_ and AUC_0–**∞**_ of four compounds were significantly increased (*p* < 0.05 and *p* < 0.01) in the UC group, symbolizing higher exposure levels in the serum of the UC state. Meanwhile, longer T_max_ and MRT_0–**∞**_ were observed for compounds 1-methoxicabony-β-carboline in the UC group (*p* < 0.05), indicating slow absorption in the pathological state. It was also found from the serum concentration-time curves that dehydroandrographolide showed double peaks in both groups, which indicated that an enterohepatic circulation might exist. The result was inconsistent with our previous literature, which may result from the difference in the composition of Chinese herbal medicines (Zhang et al., 2021). Our previous studies evaluated the pharmacokinetic profiles of Xiaoyan Lidan Formula (XYLDF) in the normal and cholestasis states. We found that some bioactive components of XYLDF have different pharmacokinetic characteristics in two kinds of physiological states, which indicated that the state of cholestasis might led to this result. Interestingly, LXF has only one less herb (xihuancao) than XYLDF, but they are used to treat different diseases in clinical. *Xihuangcao* may affect the absorption process of dehydroandrographolide. These two research were under different instrumentation and chromatographic conditions, such as the MRM in this study was in the positive ionization mode, which might cause different results.

Andrographolide and dehydroandrographolide have highly similar chemical structures. However, the C_max_, AUC_0–t,_ and AUC_0–**∞**_ of andrographolide were higher than the latter. Andrographolide has one hydroxyl group than dehydroandrographolide, which may lead to greater polarity, and the content of andrographolide is higher than dehydroandrographolide in LXF. This result is consistent with the literature (Chen et al., 2007; Xu et al., 2015). Simultaneously, 4-methoxy-5-hydroxy-canthin-6-one showed double peaks in the UC group but not showed in the control group. It is implied that the absorption and metabolism process *in vivo* might be influenced by the UC state, which is worth attention in further study.

In this study, DSS, as an inducing agent to damage gut epithelial cells and integrity of the mucosal barrier, was used to induce UC, which has been reported in the experimental animal in numerous studies (Okayasu et al., 1990; Cao et al., 2018; Dinallo et al., 2019; Li et al., 2020). UC is a debilitating and incurable disease that reduces intestinal microbes’ diversity and abundance (Bucci, 2020; Lu et al., 2022). The intestinal tract of humans is the leading site of xenobiotic metabolism, and microbes inhabiting the gastrointestinal tract can affect the metabolism outcome of environmental toxicants and pharmaceuticals, then influence their pharmacokinetics (Collins and Patterson, 2020). It might reveal the difference in the serum concentration-time curves of 4-methoxy-5-hydroxy-canthin-6-one between the control and UC groups. Meanwhile, some research shows that the microbiome regulates host gene expression, such as P450 enzymes (CYP450s), multi-drug resistance proteins, and transcription factors (Nelson, 2018; Mays and Nair, 2021). CYP450s are hemoproteins’ superfamily that catalyzes different oxidative reactions and metabolizes 70-80% of pharmaceuticals (Monserrat et al., 2020). The reduction in activities of some CYP450s may cause the serum concentration and AUC of the analytes in the UC group to increase. Comparing pharmacokinetic profiles in UC and normal states suggested that UC might change the absorption process of LXF *in vivo* and then affect its efficacy.

## 4 Conclusion

In summary, we first established a rapid, stable, sensitive, and reliable UPLC-MS/MS method to quantify the concentrations of four major bioactive components after oral administration of the LXF in rat serum. Moreover, this method was successfully applied in comparing the differences in pharmacokinetic profiles of LXF in states of physiological and UC. We found that four primary bioactive ingredients of LXF were quickly absorbed after oral administration in both states and higher exposure levels in the UC state. Collectively, the results of this research would offer some guidance in improving the therapeutic regimen and evaluating the clinical efficacy of LXF for the treatment of UC, promoting the development of personalized medicine.

## Data Availability

The original contributions presented in the study are included in the article/Supplementary Material, further inquiries can be directed to the corresponding authors.

## Abbreviations

AUC, area under the serum concentration-time curve; CE, collision energy; C_max_, maximum serum concentration; DSS, dextran sulphate sodium; IBD, inflammatory bowel disease; IS, internal standard; LLOQ, lower limit of quantification; LXF, Liandan Xiaoyan Formula; MRT, mean residence time; QC, quality control; RSD, relative standard deviation; RT, retention time; T_1/2_, elimination half-time; TCM, Traditional Chinese medicine; T_max_, maximum serum concentration; UC, ulcerative colitisPharmacokinetics of Liandan Xiaoyan Formula.
