# Supplementary figures and images for: Pharmacokinetic Study of Four Major Bioactive Components of Liandan Xiaoyan Formula in Ulcerative Colitis and Control Rats Using UPLC-MS/MS
Source: Front Pharmacol. 2022 Jul 4;13:936846. doi: 10.3389/fphar.2022.936846 (PMC9289130; doi:10.3389/fphar.2022.936846)

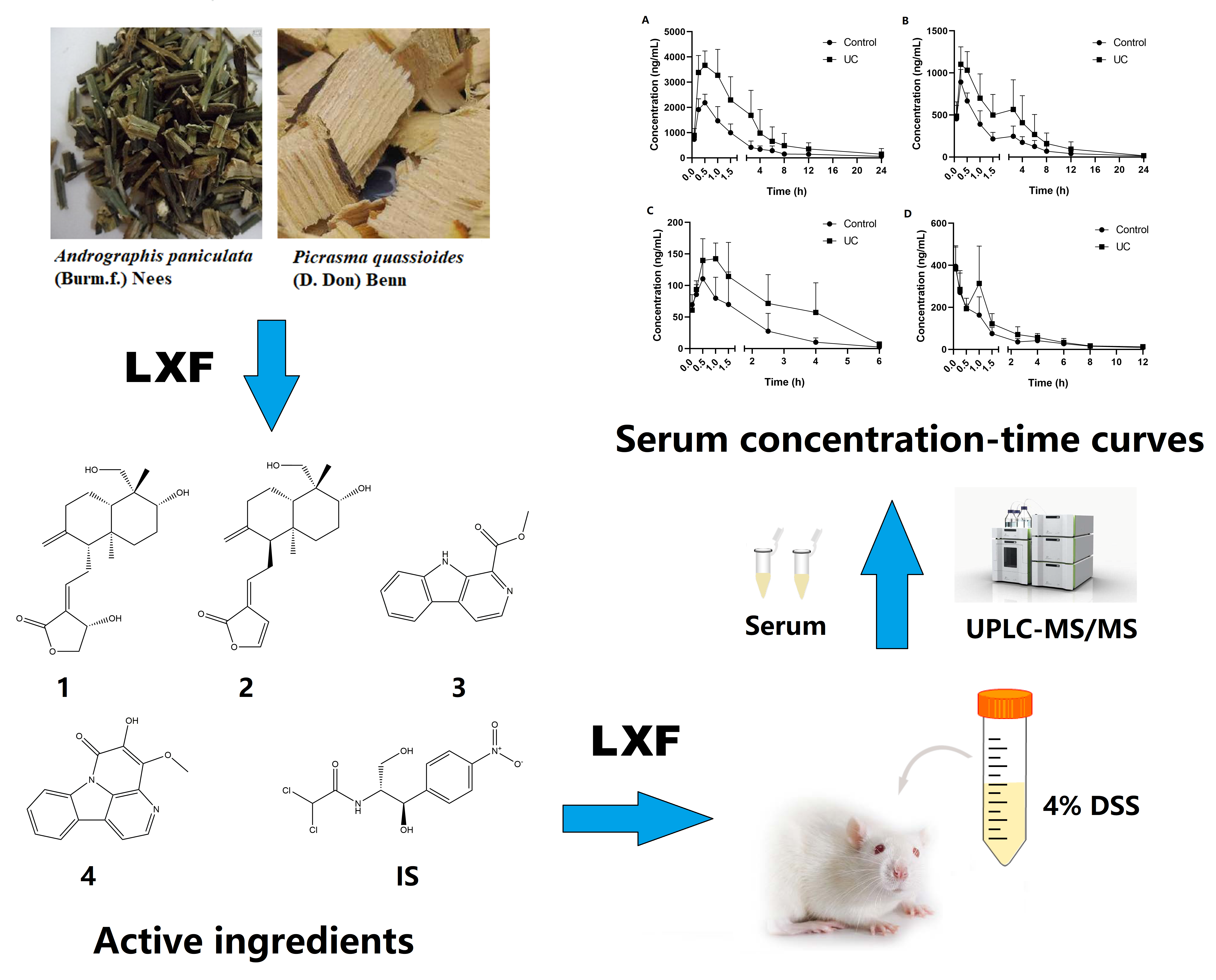

Supplement: Supplementary file 1 [file Image1.TIF]
