# Supplementary material for: Pharmacokinetic Study of Four Major Bioactive Components of Liandan Xiaoyan Formula in Ulcerative Colitis and Control Rats Using UPLC-MS/MS
Source: Front Pharmacol. 2022 Jul 4;13:936846. doi: 10.3389/fphar.2022.936846 (PMC9289130; doi:10.3389/fphar.2022.936846)
Supplement: Supplementary file 2 [file DataSheet1.docx]

Supplementary Material

# Part A: Quality control of Liandan Xiaoyan Formula (LXF)

# 1 Preparation of sample solutions

For UPLC analysis, the sample (“2.2 LXF Extract Preparation” in manuscript) was brought up to 2 mg/mL with methanol and filtered through a nylon membrane filter (0.22 μm, Phenomenex, Los Angeles, CA, USA) prior to use.

# 2 Preparation of reference solutions

Standard solutions of these four reference compounds were prepared in methanol at the known concentration (μg/mL): andrographolide (0.22 mg/mL), dehydroandrographolide (0.20 mg/mL), 1-methoxicabony-β-carboline (0.08 mg/mL) and 4-methoxy-5-hydroxy-canthin-6-one (0.20 mg/mL). All standard solutions were filtered through a nylon membrane filter (0.22 μm, Phenomenex, Los Angeles, CA, USA) before analysis.

# 3 UPLC instrumentation and methods

For quantitation of four major constituents in LXF, the UPLC was performed using Agilent 1290 Infinity II (Agilent, USA). All chromatographic separation was performed with a Zorbax Eclipse Plus C18 (100×2.10 mm, 1.8 μm, USA). The mobile phase consisted of methanol-acetonitrile (1:1) (A) and water (containing 0.1% formic acid) (B), gradient elution, at a total flow rate of 0.8 mL/min. The injection volume of all samples was 10 μL. The column was maintained at 40 ℃. UPLC Chromatogram of mixed reference substance and LXF were shown in **Supplementary Figure 1.**


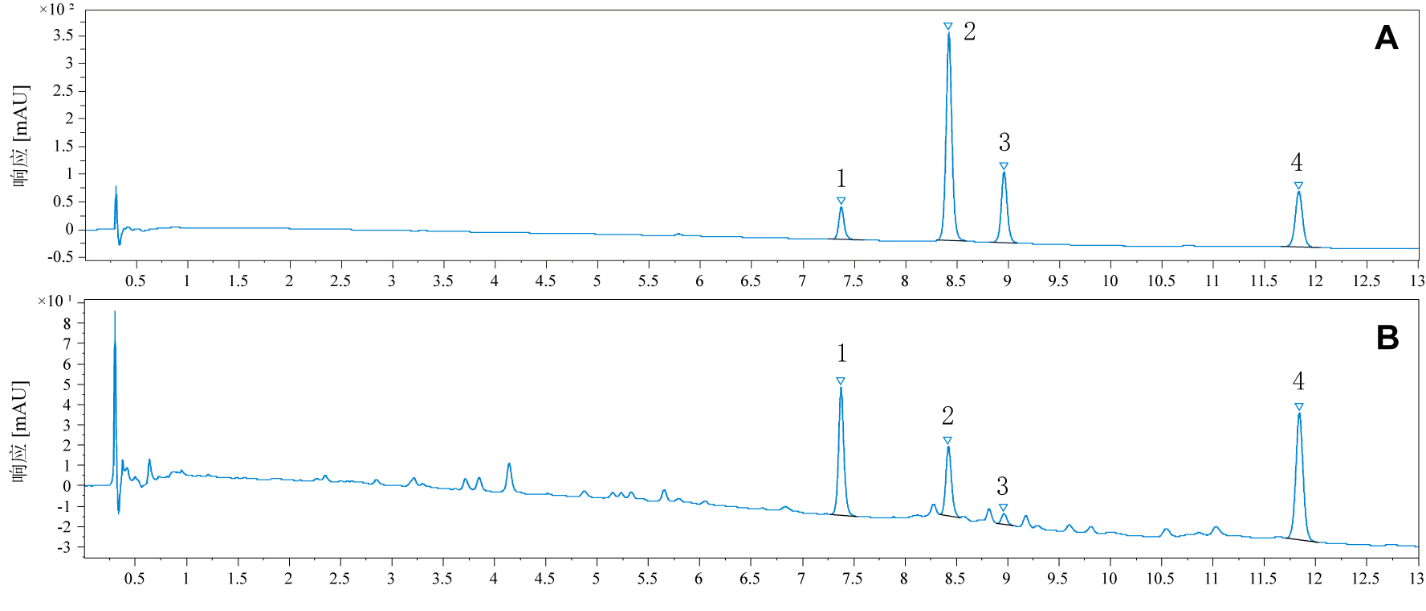
**Supplementary Figure 1** UPLC Chromatogram of a mixed reference substance (A), LXF (B)

# 4 Method validation

The linearity, ranges, regressions, LOD, LLOQ, and recoveries of the method are listed in **Supplementary Table 1**. The data exhibited a satisfactory relationship between concentrations and peak areas of the analytes within the test ranges (*R*^2^ ＞ 0.99). The RSD of repeatability, stability and standard addition recovery were not beyond 2.26%, 1.54%, 2.86%, and 1.88%, respectively. The LOD and LLOQ ranged from 0.1 to 0.5 μg/mL and 0.2 to 1.1 μg/mL, respectively. These results indicated that the developed UPLC method was efficient, accurate, and sensitive for the simultaneous quantitative determination of the four constituents in LXF.

**Supplementary Table 1** Linear-regression data, LOD, LLOQ and recovery of the four constituents determined by UPLC (*n*=6).

| **Components** | **Regression Equation** | | ***R*^2^** | **Linear Range (μg/mL)** | **LOD (μg/mL)** | **LLOQ (μg/mL)** | **Repeatability RSD (%)** | **Stability RSD (%)** | **Standard Addition Recovery RSD (%)** |
| --- | --- | --- | --- | --- | --- | --- | --- | --- | --- |
| Andrographolide | | y=21.1470x - 8.1853 | 0.9996 | 1.10-217.00 | 0.5 | 1.1 | 2.17 | 0.39 | 2.26 |
| Dehydroandrographolide | | y = 10.8880x - 4.5915 | 0.9995 | 1.00-200.00 | 0.5 | 1.0 | 1.54 | 1.50 | 1.38 |
| 1-methoxicabony-β-carboline | | y = 44.4830x - 7.4777 | 0.9993 | 0.2-80.00 | 0.1 | 0.2 | 2.86 | 1.72 | 2.58 |
| 4-methoxy-5-hydroxy-canthin-6-one | | y = 6.1724x – 3.9075 | 0.9992 | 1.00-200.00 | 0.5 | 1.0 | 1.49 | 0.79 | 1.88 |

# 5 Results

The contents of andrographolide, dehydroandrographolid, 1-methoxicabony-β-carboline, and 4-methoxy-5-hydroxy-canthin-6-one in LXF were 69.78 mg/g, 38.17 mg/g, 0.79 mg/g, and 5.68 mg/g respectively.

# 6 Conclusion

The quality of the LXF sample was controlled by the contents of the main components determined by UPLC as 69.78 mg/g of andrographolide, 38.17 mg/g of dehydroandrographolid, 0.79 mg/g of 1-methoxicabony-β-carboline, and 5.68 mg/g of 4-methoxy-5-hydroxy-canthin-6-one.

# Part B: The evaluation of the ulcerative colitis (UC) rat model establishment

Before the pharmacokinetic study, DSS-induced UC rats were confirmed to be established successfully through pharmacological indicators.

# 1 Grouping and administration

Sixteen male Sprague-Dawley rats (180–200 g) were divided into three groups: the control group and the DSS-induced (model) group (n=8). The model group were given 4% DSS for seven days, while the control group was given pure water. At the eighth day, rats were anaesthetized and for blood sample collection. Then the rats were sub-sequently sacrificed by cervical dislocation. Finally, the levels of colon length and IL-6, and IL-10 in serum were measured by combing the pathological observation to confirm the establishment of UC.

# 2 Results

As shown in **Supplementary Figure 2**, the colon length was significantly decreased (*P* < 0.01). Meanwhile, the level of IL-6 in the serum of the model group was remarkably increased, and IL-10 showed an opposite tendency (*P* < 0.05 and *P* < 0.01). The pathological observation indicated that the model group showed apparent pathological changes compared with the control group.


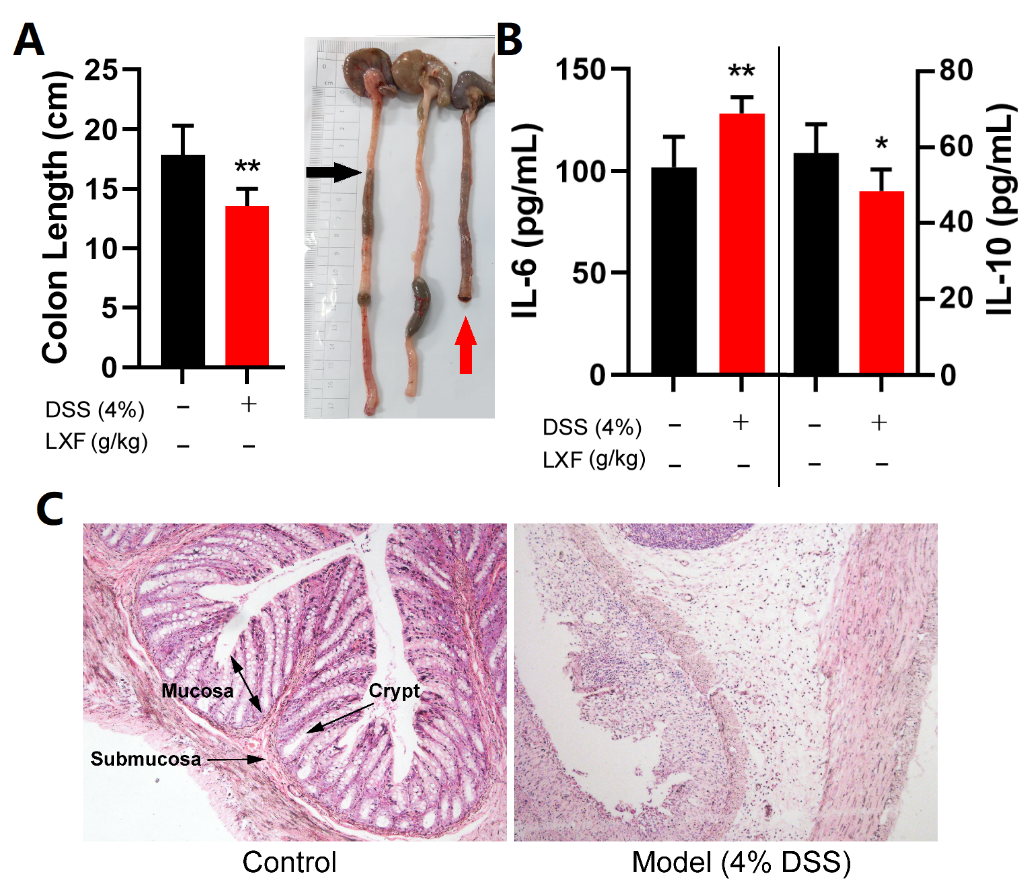


**Supplementary Figure 2** **A**: colon length. The black arrow represents for control group, and the red arrow represents for model group.; **B**: the level of IL-6, IL-10; **C**: Histology of the colon (HE,100×). ^*^ *P* < 0.05 and ^**^ *P* < 0.01 vs. the control group.

# 3 Conclusion

The model of UC was established successfully.
